# ncRNAs Orchestrate Chemosensitivity Induction by Neddylation Blockades

**DOI:** 10.3390/cancers16040825

**Published:** 2024-02-18

**Authors:** Andrea Pérez-González, Ivonne Ramírez-Díaz, Josué Guzmán-Linares, Pouya Sarvari, Pourya Sarvari, Karla Rubio

**Affiliations:** 1 International Laboratory EPIGEN, Consejo de Ciencia y Tecnología del Estado de Puebla (CONCYTEP), Instituto de Ciencias, Ecocampus, Benemérita Universidad Autónoma de Puebla (BUAP), Puebla 72570, Mexico; andrea.pg1191@gmail.com (A.P.-G.); ivonne.ramirez@upaep.edu.mx (I.R.-D.); josue.guzl98@gmail.com (J.G.-L.); 2 Faculty of Biotechnology, Popular and Autonomous, University of Puebla State (UPAEP), Puebla 72410, Mexico; 3 Iran National Elite Foundation (INEF), Tehran 1461965381, Iran; pouyasarvari2008@gmail.com (P.S.); pourya.sarvari@googlemail.com (P.S.)

**Keywords:** chemosensitivity, epigenetic modulation, neddylation, ncRNA, lung adenocarcinoma, stemness

## Abstract

**Simple Summary:**

Lung cancer is one of the leading cancers worldwide, with a poor prognosis and high mortality. Recent studies show that epigenetic mechanisms such as neddylation contribute to compromised cellular differentiation. Lung cancer cells largely exposed to conventional chemotherapy, mainly EGFR-tyrosine kinase inhibitors (TKI) (e.g., gefitinib, erlotinib, and afatinib), acquire stem-like properties that contribute to chemoresistance and tumor relapse in vitro and in vivo. Therefore, the inhibition of neddylation appears to be crucial for the understanding of the underlying reprogramming events driving tumorigenesis and an attractive epigenetics-based approach to anticancer therapies.

**Abstract:**

We performed an integrative transcriptomic in silico analysis using lung adenocarcinoma A549 cells treated with the neddylation inhibitor MLN4924 and the gefitinib-resistant PC9 cell line (PC9GR). We focused on the transcriptional effects of the top differentially expressed ncRNA biotypes and their correlating stemness factors. Interestingly, MLN4924-treated cells showed a significant upregulation of mRNAs involved in carcinogenesis, cell attachment, and differentiation pathways, as well as a parallel downregulation of stemness maintenance and survival signaling pathways, an effect that was inversely observed in PC9GR cells. Moreover, we found that stemness factor expression could be contrasted by selected up-regulated ncRNAs upon MLN4924 treatment in a dose and time-independent manner. Furthermore, upregulated miRNAs and lncRNA-targeted mRNAs showed an evident enrichment of proliferation, differentiation, and apoptosis pathways, while downregulated ncRNA-targeted mRNAs were implicated in stem cell maintenance. Finally, our results proved that stemness (*KLF4* and *FGFR2*) and epithelial–mesenchymal transition (*ZEB2*, *TWIST2*, *SNAI2*, *CDH2*, and *VIM*) factors, which are highly expressed in PC9GR cells compared to gefitinib-sensitive PC9 cells, could be abrogated with the neddylation inhibitor MLN4924 mainly through activation of epithelial differentiation pathways, thus exerting a protective role in lung cancer cells and chemosensitivity against lung tumorigenic transformation.

## 1. Introduction

Lung cancer remains the leading cause of global cancer-related death, accounting for the highest mortality rates among both men and women (https://www.who.int/news-room/fact-sheets/detail/lung-cancer, accessed on 24 November 2023). Lung cancer is usually detected only at a late stage as most patients have no obvious symptoms at the early stages of the disease [1,2,3]. Consequently, early implementation of screening programs has been proposed as one of the main tools for patients to reduce lung cancer-associated mortality [4,5,6]. Nevertheless, lung cancer has one of the lowest long-term survival rates and most lung cancers are metastatic at diagnosis, which in turn contributes to patients’ low overall survival rates [7,8,9]. Lung cancers are generally divided into two main histopathological groups based on the cell of origin: non-small cell lung cancer (NSCLC) and small-cell lung cancers (SCLC). SCLC accounts for 15–20% of total lung cancer cases, which is strongly associated with cigarette smoking and diagnosed mostly at an advanced stage [10,11]. SCLC is characterized by aggressive progression due to high cellular proliferation and early metastasis, with a still very poor prognosis; hence, it remains the deadliest of all lung cancer types [12,13,14]. The high mortality rate of SCLC patients is mainly due to the invariable development of resistance to standard chemo/radiotherapies, emphasizing the need for new therapeutic strategies [15]. NSCLC accounts for around 85% of lung cancers, which can be classified into three main subtypes: squamous cell carcinoma (SqCC), adenocarcinoma (AC), and large-cell carcinoma (LCC) [16,17]. Among them, AC is the most common histological subtype of lung cancer, accounting for approximately 40% of all lung cancers [18]. It is the most common subtype in people who have never smoked [19]. Recent research has been focused on understanding the genetic determinants of lung cancer for the identification of novel therapeutic targets and more effective treatment [20]. Overall, the NSCLC genetic landscape is complex and heterogeneous.

Resistance to chemotherapy is related to the presence of cancer stem cells (CSCs). Stem cells are undifferentiated cells that have the unique ability to self-renew, differentiate into various cell types, and give rise to a heterogeneous population of cancer cells within a tumor that also contribute to tumor onset, expansion, recurrence, and metastasis [21]. Recent research has been focused on understanding the genetic determinants of lung cancer for the identification of novel therapeutic targets and more effective treatment [20]. Specifically, new pharmacological agents targeting CSC-associated signaling pathways and surface biomarkers have been developed [22]. Moreover, it is known that the cellular and molecular constituents of CSCs also encompass epigenetic modifications that enable and promote their preservation [23].

The process of protein neddylation is abnormally activated in several types of human cancers, which makes it a good candidate for effective anticancer treatments [24,25,26,27,28,29]. Neddylation is a ubiquitination-like protein (UBL) modification characterized by the addition of NEDD8 (neural precursor cell expressed, developmentally regulated 8) to single or multiple lysine residues of the substrate protein [30,31]. Nevertheless, distinct from ubiquitination, which mostly aims for proteins destined to be degraded, neddylation modifies proteins’ function and stability [31,32]. The outcome of neddylation inhibition has been referred to as cell autophagy, cell senescence, cell apoptosis, and eventually cancer suppression [31,33]. Several other studies have revealed that the inhibition of neddylation can alleviate steatosis [34], reduce liver fibrosis [35], restrain HBV survival, and suppress pro-tumor inflammation [36]. The neddylation process requires a three-step enzymatic reaction, involving NEDD8-activating enzyme E1 (NAE1), NEDD8-conjugating enzyme E2s (UBC12/UBE2M or UBE2F), and substrate-specific NEDD8-E3 ligases [24]. One of the most potent inhibitors of neddylation is MLN4924 (Pevonedistat), which has been proven to be a powerful and specific inhibitor of NAE1 that effectively suppresses the neddylation of all cullins, causing the accumulation of their substrates [37,38,39]. Furthermore, MLN4924 was demonstrated to possess antitumor properties by inhibiting proliferation, migration, and invasion of cancer cells as well as inducing their apoptosis or senescence and autophagy in vitro and in vivo [40,41,42,43,44]. Apoptosis induced by the pharmaceutical inhibition of neddylation using MLN4924 was shown to suppress tumor growth in a variety of cancers including leukemia [45,46,47], urothelial carcinoma [40], colorectal cancer [48], and Ewing sarcoma [49]. In addition, MLN4924 exhibited anti-tumor effects via the suppression of angiogenesis in various cancer cell types including cervical cancer (HeLa), renal cell carcinoma (Caki-2), human urothelial cell carcinoma (BFTC-905), and pharyngeal squamous cell carcinoma (FaDu) cells [50]. Hence, MLN4924 holds potential for cancer treatment and has entered into several phase I clinical trials for metastatic melanoma, relapsed/refractory multiple myeloma or lymphoma, and advanced solid tumors due to its significant anticancer efficacy in preclinical studies [51,52,53]. 

Recent studies show that epigenetic mechanisms such as neddylation contribute to compromised cellular differentiation, for example, by direct regulation of histone deacetylase levels and activity [54]. Lung cancer cells largely exposed to conventional chemotherapy, mainly EGFR-tyrosine kinase inhibitors (TKI) (e.g., gefitinib, erlotinib, and afatinib), acquire stem-like properties that contribute to chemoresistance and tumor relapse in vitro and in vivo. Therefore, the inhibition of neddylation appears to be crucial for the understanding of the underlying reprogramming events driving tumorigenesis, as well as an attractive epigenetics-based approach to anticancer therapies. Our work aims to characterize the global transcriptomic profile upon pharmacological neddylation inhibition in lung cancer cells and to identify putative signaling pathways containing stemness-related mRNA panels associated with cell transformation.

Advances in whole genome sequencing (WGS) have enabled high throughput identification of genetic abnormalities including mutations, copy number aberrations, and structural alterations such as gene fusions and chromosomal rearrangements in a genome-wide unbiased manner. Extensive research performed in the last few years has resulted in the identification of genetic abnormalities that could rationalize the mechanisms of drug resistance and would better characterize NSCLC and other neuroendocrine lung tumors. However, progress in understanding the molecular basis and the biology of NSCLC has not been efficiently translated into corresponding clinical progress. This poor outcome is partly due to the high complexity of lung cancer genomes as well as the heterogeneity of lung tumors at the cellular level, with sub-clones exhibiting different combinations of gene mutations, non-coding RNA levels as well as alternative splicing gene variants. In the meantime, a better understanding of NSCLC at the molecular level requires improvements in silico techniques of multi-omics analysis, which would lead to the identification of subsets of patients that may benefit from treatment with targeted therapies.

## 2. Materials and Methods

### 2.1. RNA Sequencing Meta-Analysis

We performed an in silico analysis from published RNAseq data of lung adenocarcinoma A549 cells (*n* = 12) treated for 8 h or 24 h with 0.25 µM or 0.5 µM of the neddylation inhibitor MLN4924 (MLN, Pevonedistat, GSE134190). A second set of RNA-seq data was analyzed from the gefitinib-resistant non-small cell lung cancer (NSCLC) PC9 cell line (PC9GR) compared with its parental cell line (PC9) (*n* = 12) (GSE129221) [55]. The available normalized gene count data were downloaded as fragments per kilobase million (FPKM). Expression values of zero were set to the overall minimum value and all data were log2-transformed. Genes expressed in log2 transformed expression >0.2 were included in the analysis and measured using Pearson’s correlation. Overlapping and/or top-up (FC > 3) or top-down-regulated (FC < 0.5) genes were processed using functional enrichment pathways by KEGG, Wikipathway, and Reactome (31114916). 

### 2.2. Protein–Protein Interaction Prediction

The prediction of protein–protein interactions was conducted using the STRING online database (https://string-db.org/; accessed on 1 September 2021) [56] with a cut-off criterion of a combined score of 0.7 (high confidence) and including a maximum of 100 interactors on the 1st shell and no interactors on the 2nd shell. Network nodes represent proteins, while edges are protein–protein associations. Small nodes represent proteins of unknown 3D structure and large nodes represent proteins with some known or predicted 3D structure. Colored nodes represent query proteins and the first shell of interactors and white nodes represent the second shell of interactors. Interactions are depicted by color as follows: known interactions were obtained from curated databases (turquoise) or experimentally determined (purple); predicted interactions were defined by neighborhood (green), gene fusions (red), gene co-occurrence (blue), text mining (light green), co-expression (black), and protein homology (violet). The top Wnt signaling pathway interactors were processed using gene set enrichment analysis (GSEA) [57,58].

### 2.3. Differential Expression Analysis and Heatmaps

The mean log2-transformed FPKM expression values were processed to evaluate the transcriptomic differential expression of A549 cells treated with MLN4924 and untreated cells and PC9-Gefitinib-resistant cells (PC9GR) compared with non-resistant cells (PC9). We calculate the log2FC values and anti-log10 *p*-values to determine the levels of difference between treatments. The differential expression is shown by volcano plots graphed in RStudio (using ggplot2) using a cut-off log10 *p*-value > 0.5. Transformed gene count data were also represented in clustered heatmaps using the one minus Pearson’s correlation metric (Morpheus, Broad Institute) (Morpheus, https://software.broadinstitute.org/morpheus, accessed on 1 September 2021). For miRNA analysis, the top 100 up and down-regulated miRNAs from A549 + MLN were selected, sorted by log2foldchange from lowest to highest value, with nomenclature of mature isomiRNAs with 5p terminus, and submitted to the miRPathDB database (https://mpd.bioinf.uni-sb.de/overview.html, accessed on 1 September 2021) using the custom heatmap calculator section. The parameters used were as follows. Database: KEGG, Wikipathways, and Reactome; Evidence: Experimental (weak + strong); Select the minimum number of significant pathways a miRNA should have to be shown: 1; Select the minimum number of significant miRNAs a pathway should have to be shown: 3 (this parameter was adjusted exclusively to obtain pathways with a higher number of implicated miRNAs). 

### 2.4. Kaplan–Meier (KM) Survival Curves

The selected cohorts of lung adenocarcinoma samples included 2166 specimens (GSE102287, GSE14814, GSE157011, GSE19188, GSE29013, GSE30219, GSE31210, GSE3141, GSE31908, GSE37745, GSE43580, GSE4573, GSE50081, GSE68465, and TCGA) with available overall survival data; 62.5% of patients were male. The adenocarcinoma cohort encompassed 1161 specimens, 48.7% of whom were male. Patients were split by median survival. The chemotherapy cohort of lung adenocarcinoma samples included 173 specimens of which 125 included available overall survival data and 46.4% of patients were male. Univariate Cox regression analysis was utilized to calculate differential survival rates for each gene independently. To prevent the possibility of missing correlations because of the application of a particular cut-off, all available cut-off values between the lower and upper quartiles of expression were considered for each gene. Additional clinical variables and histology were not included in the analysis.

### 2.5. Statistical Analysis

The data for all the plots presented in the article, including the values for statistical significance and the implemented statistical tests, are provided in Source Data. Further details of statistical analysis are included in the Figures and Figure Legends. Statistical analysis and graphs were performed using Excel Solver, R-Studio, and Prism 9. Data in bar plots are represented as mean ± standard deviation (mean ± SD). Two-tailed *t*-tests were used to determine the levels of difference between the groups and *p*-values for significance. *p*-values after a two-tailed *t*-test are * *p* ≤ 0.05, ** *p* < 0.01, and *** *p* < 0.001.

## 3. Results

### 3.1. Neddylation Inhibition Reduces Stem Cell Maintenance and Survival in Lung Cancer Cells

**Figure 1:**
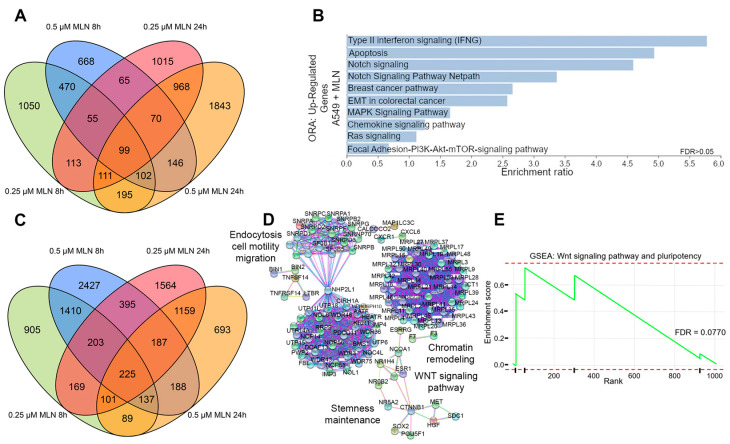
Comparative transcriptional analysis reveals reduced stemness maintenance and survival by neddylation inhibition in lung cancer cells. (**A**) Overlapped up-regulated genes (FC > 3) in A549 cells treated with MLN4924. (**B**) Over-representation analysis (ORA) of overlapping up-regulated genes in (**A**). (**C**) Overlapped down-regulated genes (FC < 0.05) in A549 cells treated with MLN4924. (**D**) Protein–protein interaction STRING network of proteins corresponding to down-regulated transcripts in (**C**). (**E**) Gene set enrichment analysis (GSEA) using the fold change values (FC < 0.05) of genes inside the Wnt and pluripotency signaling pathways in S1E. FDR, false discovery rate.

Initially, we aimed to identify differentially expressed genes (DEGs) in A549 lung cancer cell datasets retrieved from the GEO public repository. Cells were treated with 0.25 µM or 0.5 µM of the neddylation inhibitor MLN4924, either for 8 h or 24 h, respectively (Figure 1). These concentrations were chosen based on previous studies, which concluded that the application of MLN4924 on A549 and Lewis lung carcinoma (LLC) cells could significantly inhibit their proliferation, migration, and motility in a dose-dependent manner (0.1 μM, 0.33 μM, and 1 μM) [59]. In addition, they demonstrated that the application of MLN4924 (1 μM) for 15 min and 1 h could efficiently inhibit the neddylation pathway, suppress CRL activity, and cause accumulation of CRL substrates in lung cancer cells. The correlation analysis of these RNAseq-based transcriptomes allowed us to identify a positive correlation and consistent global transcription profiles between both doses within the different time points (Figure S1A,C). Therefore, we focused on the consistent 289 upregulated transcripts (4.15%, Source Data S1) with 0.25 µM and 0.5 µM MLN4924 at both time points (Figure 1A). Interestingly, over-representation analysis (ORA) revealed a significant enrichment of protein-coding genes (FC ≥ 3; Source Data S1) involved in carcinogenesis, cell attachment, and differentiation processes such as apoptosis, Notch, Ras, mTOR, and cadherin signaling pathways (Figure 1B and Figure S1B,D) upon MNL4924 treatment. 

Of note, transcript downregulation was greater compared with upregulation (10.25%; Figure 1C and Figure S1C, Source Data S1). Moreover, we extracted the top 10% of the most downregulated transcripts from the common 1010 obtained transcripts (FC ≤ 0.5; Figure S1C, Source Data S1) upon MNL4924 treatment (Figure 1C) for a network STRING analysis focused on protein-coding genes. We observed the significant enrichment of pathways related to stemness maintenance such as cell motility, migration, and Wnt signaling, therefore indicating their potential suppression by neddylation inhibition (Figure 1D and Figure S1E). In addition, these observations were further verified by gene set enrichment analysis (GSEA) of downregulated genes that showed a significant association to Wnt signaling and the pluripotency pathway (FDR = 0.0770) (Figure 1E), as well as to cellular responses to stress (ES = 0.568, *p* = 0.001, FDR = 0.0159), cell–cell communication (ES = 0.754, *p* = 0.008, FDR = 0.0275), cell cycle (ES = 0.529, *p* = 0.000, FDR = 0.0240), and PI3K-AKT (ES = 0.643, *p* = 0.005, FDR = 0.0730) (Figure S1F).

### 3.2. Reduced Cellular Stemness Correlates with ncRNA Differential Expression upon Neddylation Inhibition

Next, we performed a cross-analysis with a transcriptomic dataset using RNA sequencing from a gefitinib-resistant lung cancer cell line (PC9GR) and its parentally sensitive cells (PC9) (GSE129221) [60]. This analysis was performed to identify molecular regulators underlying neddylation-related transcriptional changes that could be associated with stemness maintenance and chemotherapy resistance in lung cancer cells. We further analyzed short non-coding RNAs (sncRNAs: miRNAs, snRNAs, and snoRNAs), long non-coding RNAs (lncRNAs), and protein-coding RNAs (mRNAs) differentially expressed in A549 lung cancer cells treated with MLN4924 compared with PC9GR cells. 

**Figure 2:**
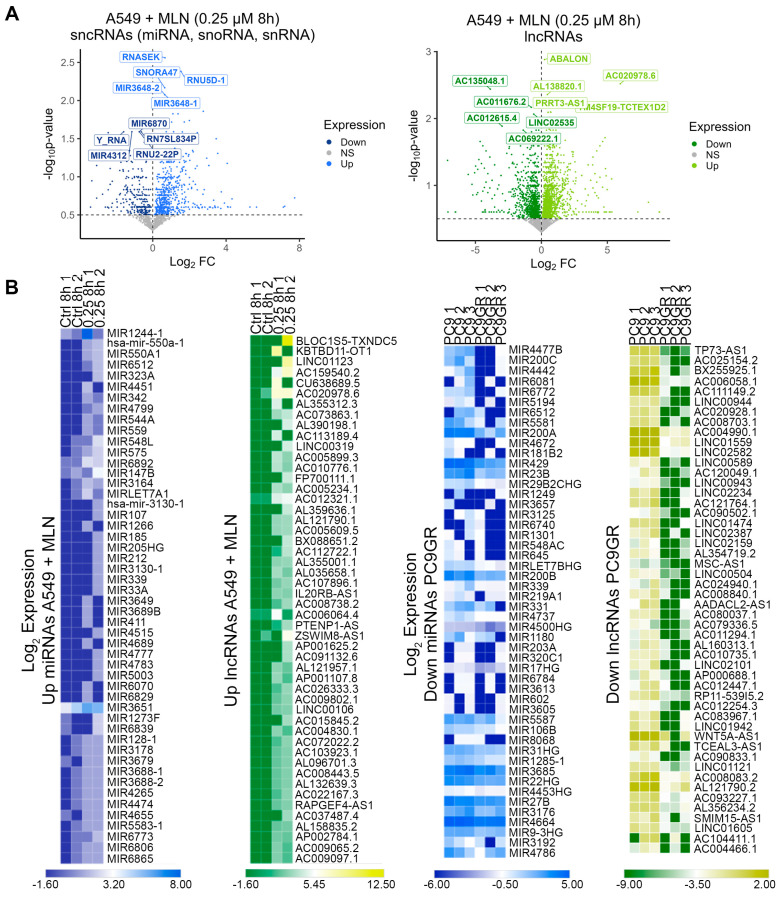
The ncRNA expression profile upon neddylation inhibition is associated with reduced cellular stemness. (**A**) Differentially expressed miRNAs, snoRNAs, snRNAs (blue), and lncRNAs (green) between A549 cells treated with MLN4924 and untreated cells. (**B**) Heatmaps of the top 50 up/down miRNAs (blue) and top 50 up/down lncRNAs (green) from the lung A549 cells treated with MNL4924 compared with the control and PC9GR cells compared with parental cells using FPKM values.

We identified the MLN4924-induced upregulation of 1497 sncRNAs (62.5%) and 2301 lncRNAs (57.8%), with concomitant downregulation of 908 sncRNAs (37.5%) and 1680 lncRNAs (42.2%) (Figure 2A, Source Data S2). In terms of mRNA expression, 58.3% of them showed significant downregulation (Figure S2B). Next, the top 50 down- and up-regulated genes were extracted (FC < 0.5 and ≥3, respectively; Source Data S2) independently in A549 cells upon MNL4924 treatment and PC9GR cells compared with their corresponding control conditions. We observed a complementary transcript regulation in PC9GR cells contrasted to the profile shown by A549 cells treated with MLN4924 (Figure 2B and Figure S2A). Based on our findings, we hypothesized that inhibiting the expression of the most significant deregulated ncRNAs upon neddylation could correlate with the production of stemness-related transcripts in the same cells.. For that purpose, we extracted the top 20 up- or down-regulated ncRNAs upon MNL4924 treatment at 8 h and 24 h. 

**Figure 3:**
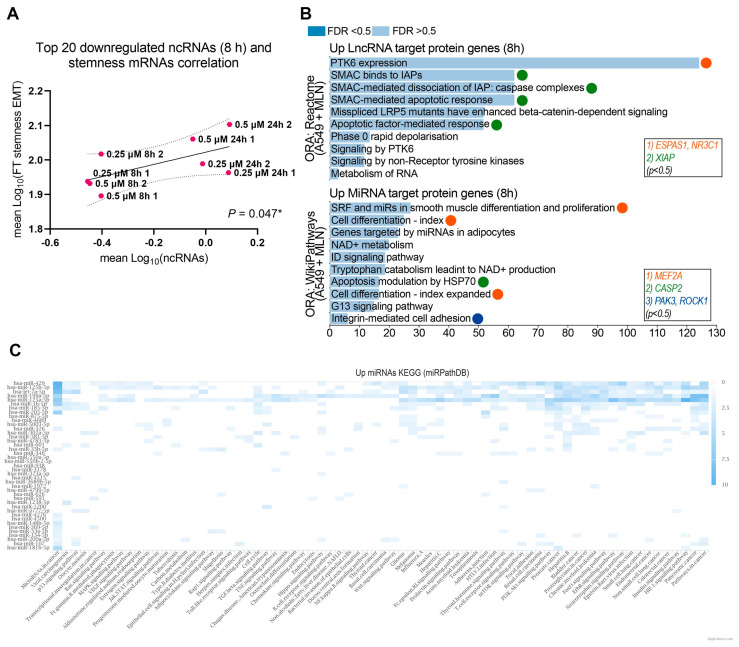
The ncRNA expression profile upon neddylation inhibition is associated with reduced cellular stemness. (**A**) Pearson correlation analysis of downregulated lncRNA expression with mRNAs expression, coding for stemness-EMT proteins in A549 cells upon MNL4924 treatment for 8 h. 2 biological replicates were included. * *p* ≤ 0.05. (**B**) Over-representation analysis (ORA) of corresponding upregulated mRNA targets of downregulated miRNAs (Wikipathway) and downregulated lncRNAs (Reactome) in A549 MNL4924-treated cells for 8 h. (**C**) Heatmap analysis (miRPathDB) of up-regulated miRNAs related to pathways in the KEGG database. In total, 41 miRNAs (*y*-axis) related to any pathway in the KEGG database (*x*-axis) are shown.

Next, we observed a significant correlation between the upregulated ncRNAs at 24 h (r = 0.964; *p* = 0.0001) (Figure S2C) and the downregulated ncRNAs at 8 h (r = 0.632; *p* = 0.047) (Figure 3A, Source Data S2). Therefore, we searched for each up- and down-regulated lncRNAs and miRNAs and their corresponding target protein-coding genes. We performed an ORA analysis using the Wikipathway-cancer, KEGG, and Reactome databases. The results showed that down-regulated ncRNAS-associated protein-coding genes were linked to stemness maintenance-related signaling pathways (Figure S2D), while those associated with up-regulated ncRNAs were linked to differentiation-related processes (Figure 3B). Overall, the MNL4924 treatment could attenuate the chemoresistance profile in lung cancer cells by upregulating specific ncRNAs that inhibit protein-coding genes related to stemness maintenance signaling (*DGCR8*, *RAD9B*, *ADAM10*, *DLK2*, *GLI3*, *GPR161*, *ANAPC10*, *CDKN2C*, and *SMC3*) and also by the downregulation of certain ncRNAs to allow the function of protein-coding genes related to cell differentiation (*MEF2A*, *EPAS1*, and *NR3C1*), cell adhesion (*PAK3* and *ROCK1*), and apoptosis (*CASP2* and *XIAP*) signaling pathways (Figure 3B). In order to bring forward the causality of miRNA dysregulation on the expression of target genes that ultimately contribute to the stem cell phenotype in lung cancer cells, we selected the top 100 up- and down-regulated miRNAs from A549 cells treated with MLN4924 (Supplementary Figure S1, Figure 2B), sorted by log2 Fold Change, from lowest to highest value, and submitted them to the miRPathDB database (Figure 3C). A KEGG enrichment analysis validated that the up-regulated miRNAs associate with pathways such as microRNAs in cancer, transcriptional misregulation in cancer, the MAPK signaling pathway, and small and non-small cell lung cancer.

### 3.3. A Neddylation Inhibitor Attenuates Therapy-Induced Survival Activated by Chemoresistance in Lung Cancer Cells

**Figure 4:**
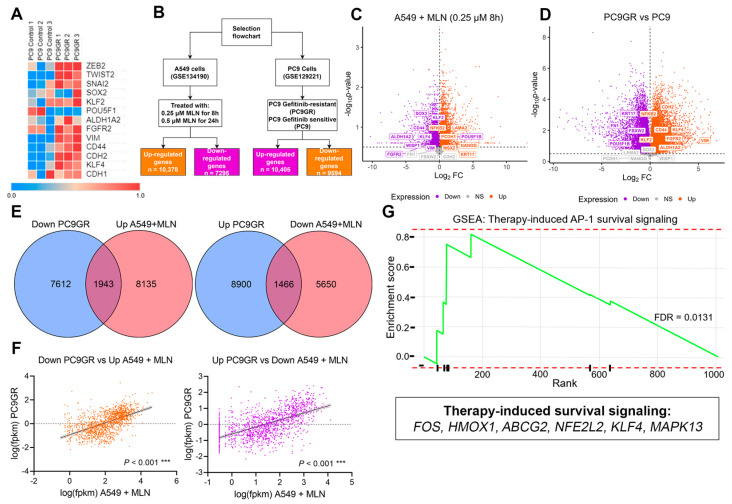
Complementary transcriptional signatures between chemoresistant and MLN4924-treated cells. (**A**) Heatmap using FPKM values of stemness-EMT transcripts of the pulmonary PC9GR cells compared with parental cells. (**B**) Flowchart displaying the two different cell lines and datasets used in the study as well as the downstream filtering and analysis performed for each dataset. (**C**) Volcano plot for differentially expressed genes between PC9 gefitinib-resistant cells compared with sensitive PC9 cells. (**D**) Volcano plot for differentially expressed genes between A549 cells treated with 0.25 µM MLN4924 for 8 h and Ctrl cells. (**E**) Left, overlap between upregulated transcripts upon neddylation inhibition and downregulated transcripts in gefitinib-resistant cells. Right, overlap between downregulated transcripts upon neddylation inhibition and upregulated transcripts in gefitinib-resistant cells. (**F**) Pearson correlation analysis was performed to evaluate the strength of the relationship between the expression of overlapping transcripts found in (**E**). *** *p* ≤ 0.001. (**G**) Gene set enrichment analysis (GSEA) using the fold change (FC > 3) of the upregulated transcripts in (**E**) (right). FDR, false discovery rate.

We explored the common transcriptional signatures between PC9GR cells and A549 MNL4924-treated cells with the aim of identifying the stemness and EMT-related genes as putative neddylation inhibitor targets to overcome chemotherapy resistance. Firstly, a global transcriptional distinction between PC9 and PC9GR cells was evidenced even upon treatment with a different TKI, Apatinib (Figure S3A). Furthermore, we observed the overexpression of selected stemness-related genes in PC9GR compared to parental cells (Figure 4A), even when treated with Apatinib (Figure S3B, Source Data S3). Therefore, we focused on the analysis of DEGs found in PC9GR cells (Figure 4B,D and Figure S3C) and in A549 cells treated with 0.25 µM of MNL4924 for 8 h (Figure 4B,C and Figure S3C) compared with their corresponding controls. We identified 8220 transcripts upregulated in PC9GR cells (51.89%, *n* = 15,841) compared to parental cells (Figure S3D). Interestingly, 1943 downregulated genes in PC9GR (Figure 4E, left) were significantly associated (Figure 4F, left, r = 0.5614; *p* < 0.001) with upregulated genes in A549 cells upon MLN treatment and vice versa (Figure 4E, right, Figure 4F, right, r = 0.593; *p* < 0.001). We used the FC values of the latter group of transcripts (FC > 3, <0.5; Source Data S3) to perform an ORA analysis based on the Wikipathway-cancer repository, which revealed the enrichment of oxidative stress and therapy-induced survival signaling pathways (Figure S3E).

In addition, this observation was further verified by analyzing the enrichment score by GSEA analysis of the MLN-induced downregulated genes inside the Reactome signature (Figure S3E), which was significant for the therapy-induced unfolded protein response (ES = 0.0445), regulation of PLK1 activity at the G2/M transition (ES = 0.746, *p* = 0.002), and cell cycle (ES = 0.503; *p* = 0.008). Alternatively, upregulated genes in PC9GR cells were enriched in therapy-induced unfolded protein responses (Figure S3F, ES = 0.767, *p* = 0.011) and most prominently in therapy-induced AP-1 survival signaling (Figure 4G, ES = 0.819, FDR = 0.0131). These results suggest that *FOS*, *HMOX1*, *ABCG2*, *NFE2L2*, *KLF4*, and *MAPK13*, which have been reported to be associated with therapy-induced survival signaling (Figure 4G), are the main regulators of chemoresistance in PC9GR cells and thereby could be a promising signature to promote chemosensitivity by MNL4924 treatment in lung cancer.

### 3.4. Stemness and Epithelial–Mesenchymal Transition Factors Act as Neddylation Targets to Attenuate Chemoresistance Responses in Lung Cancer

**Figure 5:**
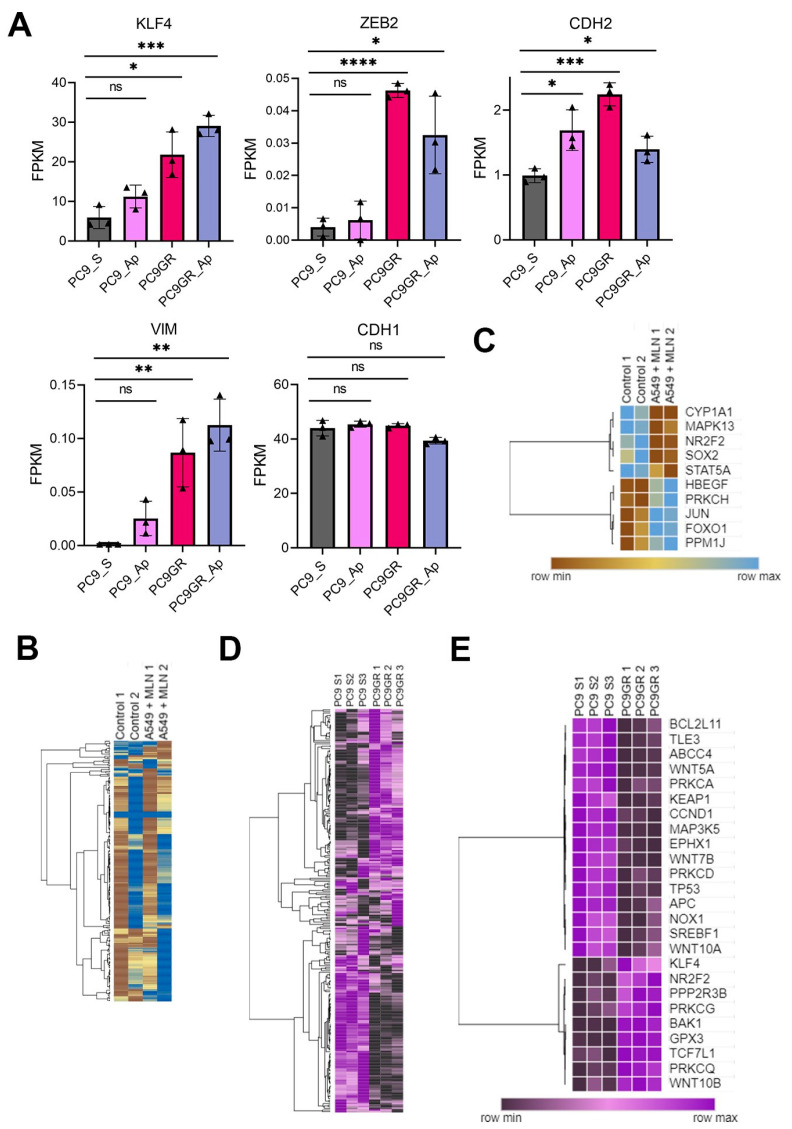
Stemness and epithelial–mesenchymal transition factors as neddylation targets in lung cancer cells. (**A**) Selected stemness-EMT target genes in PC9GR compared to the mean FPKM values in parental cells and in both treated with Apatinib (AP). Bar plots presenting data as means; error bars, s.e.m. (*n* = 3 biologically independent experiments); asterisks, *p*-values after one-way ANOVA, post hoc Dunnet: **** *p* ≤ 0.0001; *** *p* ≤ 0.001; ** *p* ≤ 0.01; and * *p* ≤ 0.05; ns, no significant. (**B**) Heat map showing differentially expressed genes (DEGs) in MNL4924 treated cells compared to the mean FPKM values of untreated cells. Columns represent replicates of each condition and rows represent individual genes. The tree next to the heatmap demonstrates the hierarchical clustering of individual samples. Blue represents up-regulated genes and brown represents down-regulated genes. (**C**) A cluster of DEGs affected by a neddylation blockade in A549 cells. (**D**) Heat map showing a comparison of all differentially expressed genes (DEGs) between PC9GR cells and gefitinib-sensitive PC9 cells using the mean FPKM values. Columns represent replicates of each condition and rows represent individual genes. The tree next to the heatmap demonstrates the hierarchical clustering of the samples. Purple represents up-regulated genes and black represents down-regulated genes. (**E**) DEGs cluster in PC9- chemoresistant cells. FPKM, fragments per kilobase per million mapped fragments.

**Figure 6:**
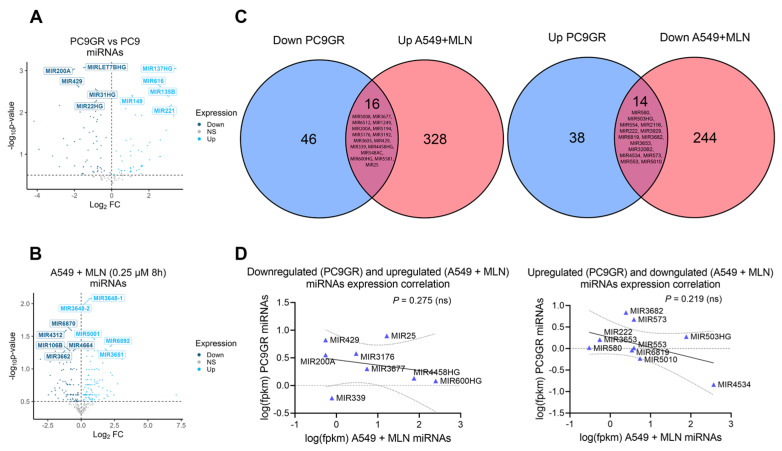
Reprogramming by MLN4924 in TKI-resistant cells is not associated with alterations in miRNA landscapes. (**A**) Differentially expressed miRNAs between PC9GR and parental cells. (**B**) Differentially expressed miRNAs between A549 cells treated with 0.25 µM MLN4924 at 8 h and untreated cells. (**C**) Overlapping DEGs coding miRNAs upregulated under neddylation inhibition and downregulated in gefitinib resistance cells (left panel). Overlapping DEGs coding miRNAs downregulated under neddylation inhibition and upregulated in gefitinib resistance cells (right panel). (**D**) A Pearson correlation analysis was performed to evaluate the strength of the relationship between the expression of overlapping genes found in (**C**).

The transcriptional profile of critical stemness-related factors was analyzed to examine whether an individualized signature was responsible for the TKIs-chemoresistance in lung cancer. We observed the overexpression of *KLF4*, *CDH2*, *VIM*, and *ZEB2* in PC9GR cells compared to gefitinib-sensitive PC9 cells (Figure 5A, Source Data S4). Hence, we interrogated if this effect was induced by miRNA dysregulation upon neddylation inhibition. For this purpose, we identified 16 overlapping miRNAs downregulated in PC9GR cells and upregulated upon MNL4924 treatment, as well as 14 overlapping miRNAs upregulated in PC9GR cells and downregulated upon MNL4924 treatment (Figure 6A–C, Source Data S5).

Nevertheless, there was only a slight negative correlation among their expression values (Figure 6D). Then, we concluded that reprogramming by MLN4924 in TKI-resistant cells might involve a stronger contribution of lncRNAs rather than miRNAs. Furthermore, through clustered heatmaps comparing global transcriptomes between A549 MNL4924-treated (Figure 5B) and PC9GR cells (Figure 5D) with their respective control conditions, we identified two DEGs clusters (Figure 5C,E, Source Data S4). 

**Figure 7:**
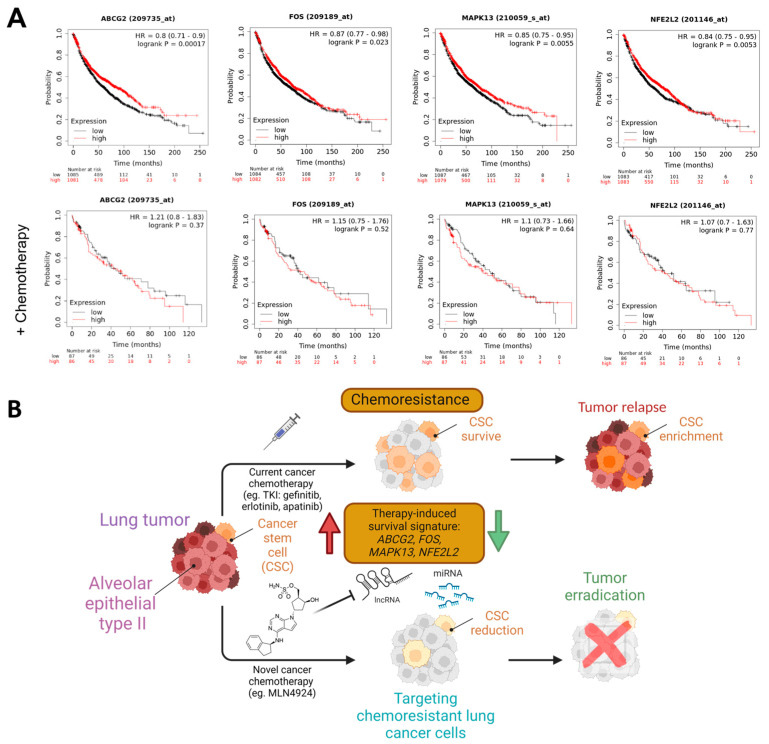
Neddylation blockades-responsive mRNAs as prognostic markers in lung cancer. (**A**) Overall survival rates by a Kaplan-Meier plotter of total (*n* = 2166) or chemotherapy-treated (*n* = 173) LUAD patients expressing low vs. high expression of therapy-induced survival signature markers. HR, hazard ratio. Created with Biorender.com. (**B**) Schematic model representing a neddylation inhibitor attenuating the therapy-induced survival signaling and tumor relapse by *ABCG2*, *FOS*, *MAPK13,* and *NFE2L2* downregulation (green arrow) that are activated in chemotherapy-resistant lung cancer cells (red arrow) induced by current chemotherapies.

Our results showed that *MAPK13* (therapy-induced survival marker) and *SOX2* (stemness maintenance-related marker) were downregulated in A549-treated cells (Figure 5C). On the other hand, *KLF4* and *NR2F2* (stemness maintenance-related markers) were overexpressed in PC9GR (Figure 5E). Furthermore, we retrieved LUAD expression data of 2166 patients from the Kaplan–Meier plotter [61] (Figure 7A). We observed that lung adenocarcinoma patients with higher levels of the therapy-induced survival signature markers *ABCG2*, *FOS*, *MAPK13,* and *NFE2L2* showed a significant higher overall survival of 86, 73, 75.43, and 80 months, respectively (HR = 0.8, *p* = 0.00017; HR = 0.87, *p* = 0.023; HR = 0.85, *p* = 0.0055; HR = 0.84, *p* = 0.0053), compared to the overall survival of 61, 63.03, 64, and 62 months of patients with low expression levels (Figure 7A, upper panels). Remarkably, this effect on patient survival is significantly affected by chemotherapy (Figure 7A, lower panels). These findings suggest the clinical relevance and protective role of neddylation inhibition in the context of chemotherapy resistance markers in lung cancer patients, which might prove helpful in developing novel therapeutic approaches for lung cancer (Figure 7B).

## 4. Discussion and Perspectives

NEDD8 is ubiquitously expressed and evolutionarily shares 100% homology among mouse, rat, and human orthologs [32,62]. NEDD8 is primarily produced as a precursor protein containing 81 amino acid residues, which is then cleaved by several proteases (NEDP1, USP21, and UCH-L3) leaving the carboxy-terminal glycine (Gly) 76 residue exposed [63,64]. Alike ubiquitination, the process in which NEDD8 conjugates to target proteins is consecutively mediated through NEDD8-specific E1 activating enzymes (NAEs) and E2 conjugated enzymes (UBC12/UBE2M and UBE2F) and several unknown E3 ligases [27]. The activation of the mature NEDD8 C-terminal residues is an ATP-dependent procedure that occurs by forming a thioester bond with E1 enzymes (NAE), an important regulator of the E3 ubiquitin ligase SCF (SKP1, cullins, and F-box protein), and is also implicated in DNA damage and repair. The activated NEDD8 is further transported from the active cysteine site of NAE to the active cysteine site of E2 conjugating enzymes. E2 conjugating enzyme then interacts with E3 ligases, resulting in the transfer of the NEDD8 and the formation of an isopeptide bond between the C-terminal glycine-76 of NEDD8 and an ε-amino group of lysine in the target protein [30,38,65]. The cullin family is widely recognized as one of the main physiological targets of neddylation. The most important member of this family is cullin-RING ligases (CRLs), which are the biggest group of E3 ligases involved in ubiquitination [31,66]. CRLs are responsible for about 20% of the degradation of cellular proteins by the proteasome.CRLs are activated by cullin neddylation, which in turn promotes the ubiquitination of substrates [31,67]. Therefore, neddylation plays an important role in regulating cellular function partially via modulating the E3 ubiquitination ligases. It has been demonstrated that inhibition of neddylation can result in the inactivation of CRLs [39], which in turn leads to the accumulation of their substrates, such as p21 [68], nuclear factor erythroid 2-related factor 2 (NRF2) [69,70], and chromatin licensing DNA replication factor 1 (CDT1) [71]. Among CRL substrates, a vast number have been reported to be tumor suppressors [72]. 

Neddylation inhibition appears to be crucial for understanding the underlying cellular reprogramming events and to be an attractive epigenetics-based approach for anticancer therapies. Previous studies have shown that MLN4924, a promising small molecule that inhibits NAE, may act as a therapy sensitizer in other cancer subtypes, such as leukemia, cervical, esophageal, ovarian, and urothelial carcinomas [37,73,74,75,76,77,78]. For instance, at nanomolar doses (20–100 nmol/L), MLN4924 selectively inhibits cullin neddylation leading to an enhancement of radiation-induced DNA damage, cell-cycle arrest, and apoptosis, which sensitizes pancreatic cancer cells as well as lung cancer cells to radiotherapy.. MLN4924 and cisplatin cooperate to induce DNA damage, oxidative stress, and increased expression of the proapoptotic protein NBK/BIK. Moreover, MLN4924 has substantial efficacy against both cisplatin-sensitive and cisplatin-resistant cells in ovarian cancer models, indicating that abnormal neddylation could play a role in the development of drug resistance [74].

In addition, MLN4924 was shown to trigger intrinsic and extrinsic cellular apoptosis through the inactivation of cullin NEDDylation, leading to an elevated level of cleaved caspase3 and cleaved caspase7 and reduced expression levels of *Bcl-2* and *Bcl-XL* in a dose-dependent manner [79,80,81] as well as induction of the NOXA-dependent apoptosis [82,83]. Moreover, MLN4924 was known to alter mitochondrial morphology, perturb mitochondrial tricarboxylic acid cycle (TCA) and glycolysis metabolites, and reprogram energy metabolism in cancer cells by promoting mitochondrial OXPHOS and glycolysis [84,85,86]. Hence, the combined treatment of MLN4924 with the glycolysis inhibitor or OXPHOS inhibitors such as metformin can potentially enhance antitumor effects both in vitro and in vivo. In this study, we found that lung cancer treatment with the neddylation inhibitor MLN4924 may induce epithelial differentiation as a protective role, inducing chemosensitivity against lung tumorigenic transformation. To characterize the global transcriptomic profile upon pharmacological neddylation inhibition and to identify putative signaling pathways associated with cell transformation, we performed an integrative in silico analysis from RNA sequencing data using lung adenocarcinoma A549 cells (lung adenocarcinoma) treated with two different concentrations of the neddylation inhibitor MLN4924 at two different time points (GSE134190). In addition, we retrieved and analyzed RNAseq data from the gefitinib-resistant non-small cell lung cancer (NSCLC) PC9 cell line (PC9GR) from GEO datasets (GSE129221) to validate the cellular effects of neddylation inhibition on therapy resistance. Across the clusters of upregulated and downregulated genes and their pathway enrichment analysis, we focused on the transcriptional effects in top differentially expressed ncRNA biotypes and stemness factors. Previous studies showed that the entire neddylation pathway was overactivated in human lung cancer cells (H1299, A549, and H460) [59]. In addition, they demonstrated that the application of MLN4924 (1 μM) for 15 min and 1 h could efficiently inhibit the neddylation pathway, suppress CRL activity, and cause the accumulation of CRL substrates in lung cancer cells. Moreover, the application of MLN4924 on A549 and Lewis lung carcinoma (LLC) cells could significantly inhibit their proliferation, migration, and motility in a dose-dependent manner (0.1 μM, 0.33 μM, and 1 μM). Moreover, MLN4924 exerted chemosensitizing effects in A549 and H460 cells treated with carboplatinum or cisplatinum, two widely-used conventional cytotoxic agents for the clinical treatment of lung cancer. Another study showed that the application of sub-toxic concentrations of MLN4924 (0.04, 0.2, or 1 μM) on a human osteosarcoma (OS) cell line, namely SJSA-1, for 6, 24, or 48 h inhibits the neddylation of cullins and blocks the degradation of CRL substrates including CDT1, p27, p21, Wee1, Noxa, p16, and cyclin E associated with cell proliferation and apoptosis. In addition, p21 levels peaked within 6 h of MLN4924 treatment and were sustained for 48 h in the presence of MLN4924. Meanwhile, the application of MLN4924 at 5 μM almost completely inhibited cell viability in SJSA-1 and MG-63 cell lines, indicating a strong cytotoxic effect of MLN4924 at high doses [87]. Another study showed that sub-toxic concentrations of MLN4924 (0, 0.5, 1, and 1.5 μM) for 12 h could inhibit the degradation of CRL substrates and induce G2/M arrest in renal cancer cells [42]. However, other studies showed that MLN4924 at a low concentration (30–100 nM) could stimulate cancer cell proliferation, sphere formation, and tumorigenesis in various human cancer cell lines, including H1299 (lung cancer), MCF7 and SUM159 (breast cancer), and HCT116 (colorectal cancer) [88]. In addition, MLN4924 at moderate concentrations (250 and 500 nM) was shown to accelerate cell migration at least in two cancer cell-lines including in a prostate cancer cell-line PC3 and in a glioblastoma cell-line U373MG after 24 h [89]. Hence, the results obtained from the application of MLN4924 should be interpreted with caution depending on the used concentration and cell contexts. 

The A549 cell line is derived from adenocarcinoma human alveolar basal epithelial cells. It has been documented to exhibit resistance to EGFR-TKI treatment [90] and is extensively employed in fundamental research for the development of anticancer drugs. [91]. The PC9 cell line, on the other hand, is a human pulmonary adenocarcinoma cell line with a deletion mutation in exon 19 of the EGFR gene that exhibits high sensitivity to TKIs [92]. The gefitinib-resistant lung cancer cell line (PC9GR) is established from its parental sensitive line (PC9) with a traditional EGFR mutation after long time exposure to gefitinib, which shows a quite different transcription, signaling, and metabolic profiles [55]. Cancer cells are known to develop resistance to EGFR TKIs after prolonged exposure, with over half of TKI-resistant cases able to be attributed to the EGFR-T790M mutation [93]. These molecular distinctions contribute to the different responses and behaviors of the A549 and PC9 cell lines in terms of growth, migration, invasion, and drug resistance. Hence, it is important to understand the molecular distinctions between A549 and PC9GR cells to develop efficacious therapeutic approaches for non-small-cell lung cancer (NSCLC). Therefore, we explored the differential expression of stemness and EMT-associated factors in PC9GR cells compared to gefitinib-sensitive PC9 cells. Interestingly, A549 MLN4924-treated cells showed upregulation of protein-coding genes (*KRT17* and *LAMA2*) involved in carcinogenesis, cell attachment, and differentiation pathways in a concentration-independent manner; with concomitant downregulation of stemness maintenance and survival signaling pathways (*SOX2*, *KLF4*, *ALDH1A2*, *FGFR2*, *CD44*, *VIM*, and *WISP1*), an opposite effect observed in PC9GR cells. Remarkably, 1943 downregulated genes in PC9GR cells were significantly associated with upregulated genes upon MLN4924 treatment. Contrarily, 1466 upregulated genes in PC9GR cells correlated significantly with downregulated genes upon MLN4924 treatment. Although the molecular differences between the A549 and PC9 cell lines significantly influence their responsiveness to therapy, the evidence shows that both cells have similar behavior for certain drugs. Both cell lines exhibited growth suppression and apoptosis induction upon treatment with Shikonin (SHK, a pleiotropic agent with remarkable cell growth inhibition activity against NSCLC and other cancer types) [94]. Furthermore, the combination of SB203580, a p38MAPK inhibitor, with gefitinib enhances its antitumor effect in PC-9 and A549 lung cancer cell lines [95]. These data indicate that SHK and SB203580 may exert comparable effects on the A549 and PC-9 cell lines. Further experimental work is necessary to fully understand the molecular similarities and differences across these cell lines, which is a limitation of this work. Nevertheless, there is a lack of conclusive data demonstrating the inhibitory effect of MLN4924 on stemness in human cells. Additionally, MLN4924 can enhance the susceptibility of certain cancer cells to tumor necrosis factor-α (TNF)-induced cell death, suggesting its capacity to suppress the stemness of cancer cells [96]. As well, MLN4924 displayed inhibitory effects on cell proliferation, migration, and invasion in human clear cell renal cancer (ccRCC) cells, indicating its potential therapeutic significance in suppressing stemness in ccRCC [42]. 

So far, MLN4924 is considered one of the most potent and the most reported NAE inhibitors with potential therapeutic effects for other diseases and disorders besides different cancer types [85]. For instance, MLN4924-induced Neddylation inhibition displays neuroprotective effects against oxidative stress injury [97]. In addition, these protective effects are associated with diminished ROS production induced by H_2_O_2_ and the accumulation of Nrf2 protein levels in the cytoplasm and nucleus of cerebellar granule neurons (CGNs). However, further research will be required to elucidate how MLN4924 mediates neuronal protection prior to clinical approaches in neurodegenerative diseases. Moreover, studies demonstrated that MLN4924 has protective roles against ischemic injury by attenuating neutrophil extravasation and maintaining blood–brain barrier integrity (BBB) [98]. Furthermore, MLN4924 exhibited strong cardioprotective effects against myocardial ischemia/reperfusion (MI/R) via induced autophagic flux and up-regulated *Nrf2* mediated by SIRT1 [99]. Overall, these findings suggest that MLN4924 poses as an effective therapeutic target not only for the treatment of cancers but also for various diseases as mentioned above. However, there are some challenges and limitations associated with NAE inhibitors including MLN4924. For instance, patients with acute myeloid leukemia (AML) and myelodysplastic syndromes (MDS), in a phase I clinical trial, showed the overall low response rate of only 17% to MLN4924 [100], ClinicalTrials.gov Identifier: NCT00911066, [85]. MLN4924 showed several side effects including tumor sphere formation and ciliogenesis inhibition through the c-MYC accumulation and dimerization of EGFR, activating the EGFR signaling pathway [88,101]. In order to improve MLN4924 antitumor potency and reduce its side effects, an optimization of MLN4924 performed using a structural hopping strategy led to the design of a compound bearing a pyrimidotriazole scaffold [102], which showed significant antitumor efficacy and good safety in xenograft models. Thus, developing novel neddylation inhibitor agents with improved anticancer efficacy, selectivity, and safety could be a potential future direction for cancer treatment. In addition, an increasing line of evidence suggests that MLN4924 coupled with different anticancer drugs can overcome drug resistance and improve anticancer results [103,104,105,106]. Hence, combined pharmacotherapy or multi-target drugs seem to be promising therapeutic approaches for the future. 

Our meta-analysis with patient expression data from different cohorts shows that our suggested mechanism of neddylation inhibition might, in consequence, impact the overall patient survival by the attenuation of therapy-resistance markers. However, we are still unable to clearly set the role of MLN4924 in the treatment of lung adenocarcinoma due to insufficient experimental data, which could be performed either by in vitro designs with lung cancer immortalized cells or with patient-derived primary cultures. Thus, the single chemotherapy versus combination regimens including neddylation inhibitors would be worth it to explore in future studies. The strength of this investigation is that it provides a systematic in silico study that provides the mechanistic insights and educated ncRNA guesses underlying a reversible phenotypical transformation in lung cancer cells displaying chemotherapy sensitivity by neddylation modulation. 

## 5. Conclusions

Our results indicate that stemness factors are inhibited by up-regulated ncRNAs after MLN4924 treatment in lung cancer cells. Mechanistically, miRNAs and lncRNAs-targeted mRNAs showed a strong association with cellular proliferation, differentiation, and apoptosis. Downregulated ncRNAS upon MLN4924 treatment showed a target preference towards mRNAs implicated in stemness maintenance. Furthermore, neddylation inhibitors attenuate therapy-induced survival markers (including *ABCG2*, *NFE2L2*, *FOS*, *HMOX*, and *MAPK13*) that are activated in PC9GR cells. Finally, we proved that stemness (*KLF4* and *FGFR2*) and epithelial–mesenchymal transition (*ZEB2*, *TWIST2*, *SNAI2*, *CDH2* and *VIM*) factors are overexpressed in PC9GR cells compared to gefitinib-sensitive PC9 cells. Our analysis suggests that lung cancer treatment with the neddylation inhibitor MLN4924 may induce epithelial differentiation as a protective role, inducing chemosensitivity against lung tumorigenic transformation. However, the suggested mechanisms through which MLN4924 induces chemosensitivity in lung cancer cells require further experimental validation to confirm its effectiveness in vitro and ex vivo models, as well as its clinical applicability in combination with TKIs.

## Data Availability

Data are contained within the article and Supplementary Materials.

## Supplementary Materials

The following supporting information can be downloaded at https://www.mdpi.com/article/10.3390/cancers16040825/s1. Figure S1: Pathway enrichment analysis of cellular stress and adhesion-related genes upon neddylation inhibition in lung cancer cells; Figure S2. Differential ncRNA expression response upon treatment with neddylation inhibitor; Figure S3. The gene-expression profiles from tumorigenic chemosensitive and parental lung cancer cells; Data S1: Transcriptional profiles of A549 cells treated with MLN4924; Data S2: Differentially expressed ncRNAs in A549 cells treated with MLN4924 compared with PC9GR cells; Data S3: Correlation analysis of DEGs in PC9GR cells and in A549 cells treated with MNL4924; Data S4: Expression of stemness factors upon neddylation inhibition in PC9GR cells compared to gefitinib-sensitive PC9 cells; Data S5: miRNA dysregulation upon neddylation inhibition in PC9GR cells compared to gefitinib-sensitive PC9 cells.
