# Supplementary material for: ncRNAs Orchestrate Chemosensitivity Induction by Neddylation Blockades
Source: Cancers (Basel). 2024 Feb 18;16(4):825. doi: 10.3390/cancers16040825 (PMC10886669; doi:10.3390/cancers16040825)
Supplement: Supplementary file 1 [file cancers-16-00825-s001.zip › Supplementary Figures S1-S3.pdf]

Supplementary Figures S1–S3

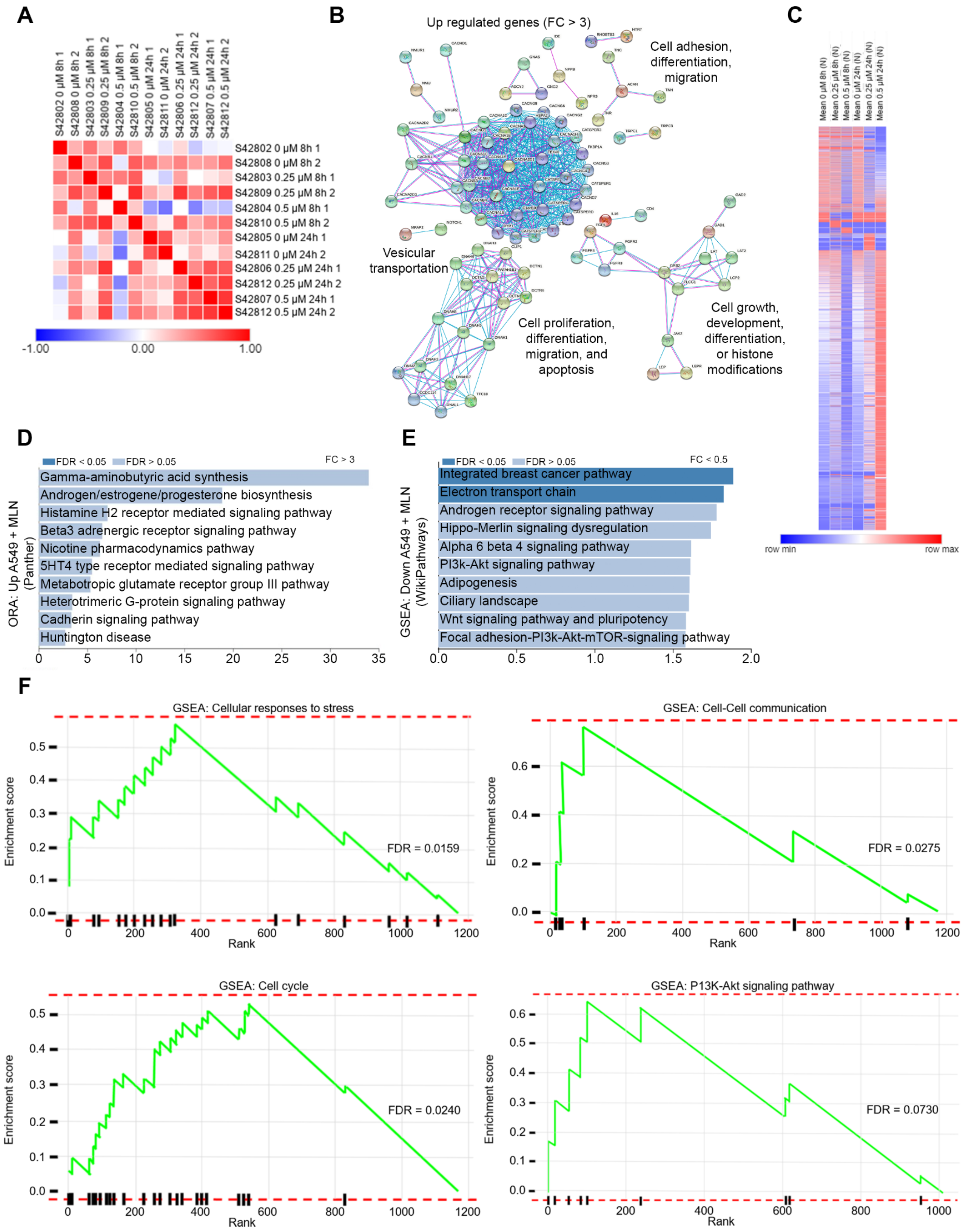

Supplementary Figure S1. Pathway enrichment analysis of cellular stress and adhesion-related genes upon neddylation inhibition in lung cancer cells (A) Heat map representing the RNA-seq-based expression levels of the total 41451 transcripts (x-axis: samples of 0.25 uM and 0.5 uM MNL at 8 h, 0.25 uM and 0.5 uM MNL at 24 h, and DMSO, n = 6; in red: upregulation, in blue: downregulation). (B) Protein-protein interaction network (from STRING) of corresponding protein-coding upregulated genes in 1A. (C) Correlogram showing the correlation score matrix (FPKM values, Kendall correlation coefficient) across RNA-seq data of lung A549 cancer cells treated with MLN4924. (D) Over-representation analysis (ORA) from Panther signaling pathways of overlapping upregulated (FC>3) genes in 1A. (E) Gene Set Enrichment Analysis (GSEA) from Wikipathway signaling pathways of overlapping downregulated (FC0.5) genes in 1C. (F) Gene Set Enrichment Analysis (GSEA) using the fold change of genes inside the cellular response to stress, cell-cell communication, cell cycle, and PI3K-Akt pathways. FDR, False discovery rate.

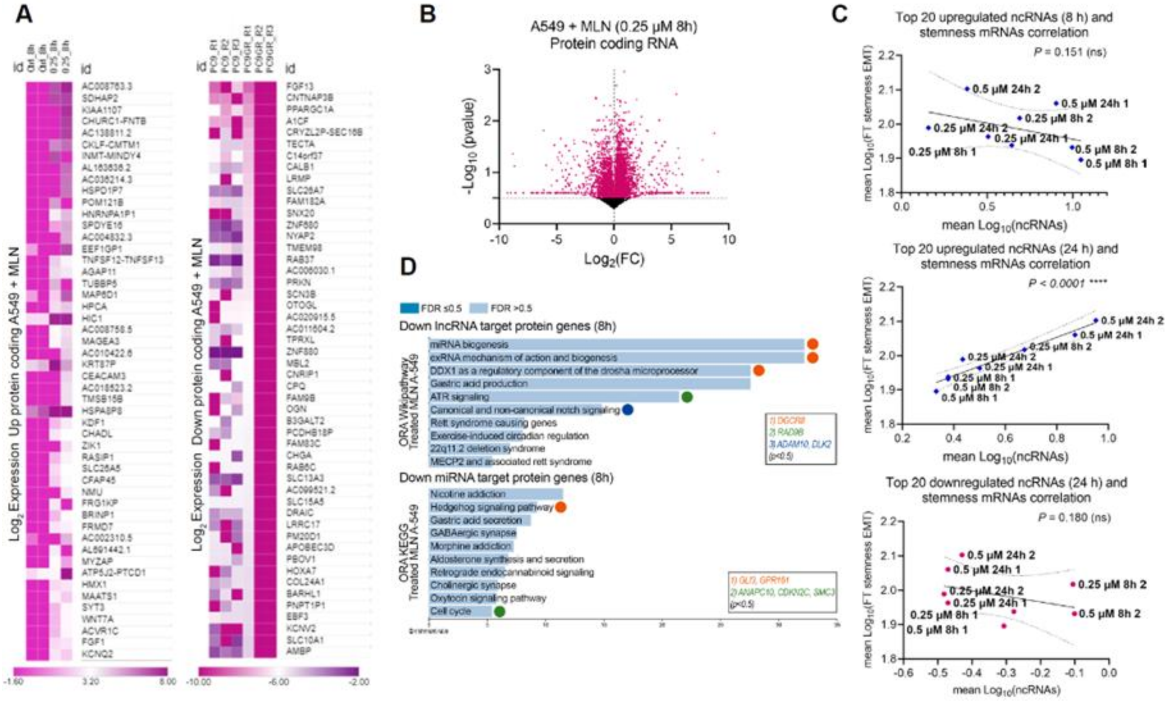

Supplementary Figure S2. Differential ncRNA expression response upon treatment with neddylation inhibitor (A) Pearson correlation analysis was performed to evaluate the strength of the relationship between the expression of the top 20 up- and down-regulated ncRNAs and mRNAs coding for stemness-EMT proteins in A549 cells under MNL4924 treatment at both 8h and 24h. (B) Differentially expressed mRNA genes in A549 cells treated with MLN4924 compared to untreated cells. (C) Heatmaps of the top 50 upregulated mRNAs (in MNL4924 treatment) and the top 50 downregulated mRNAs (in PC9GR cells) compared with their respective control conditions using FPKM values. (D) Over-representation analysis (ORA) of corresponding mRNA targets of upregulated miRNAs (KEGG) and upregulated lncRNAs (Wikipathway) in A549 MNL4924-treated cells at 8h.

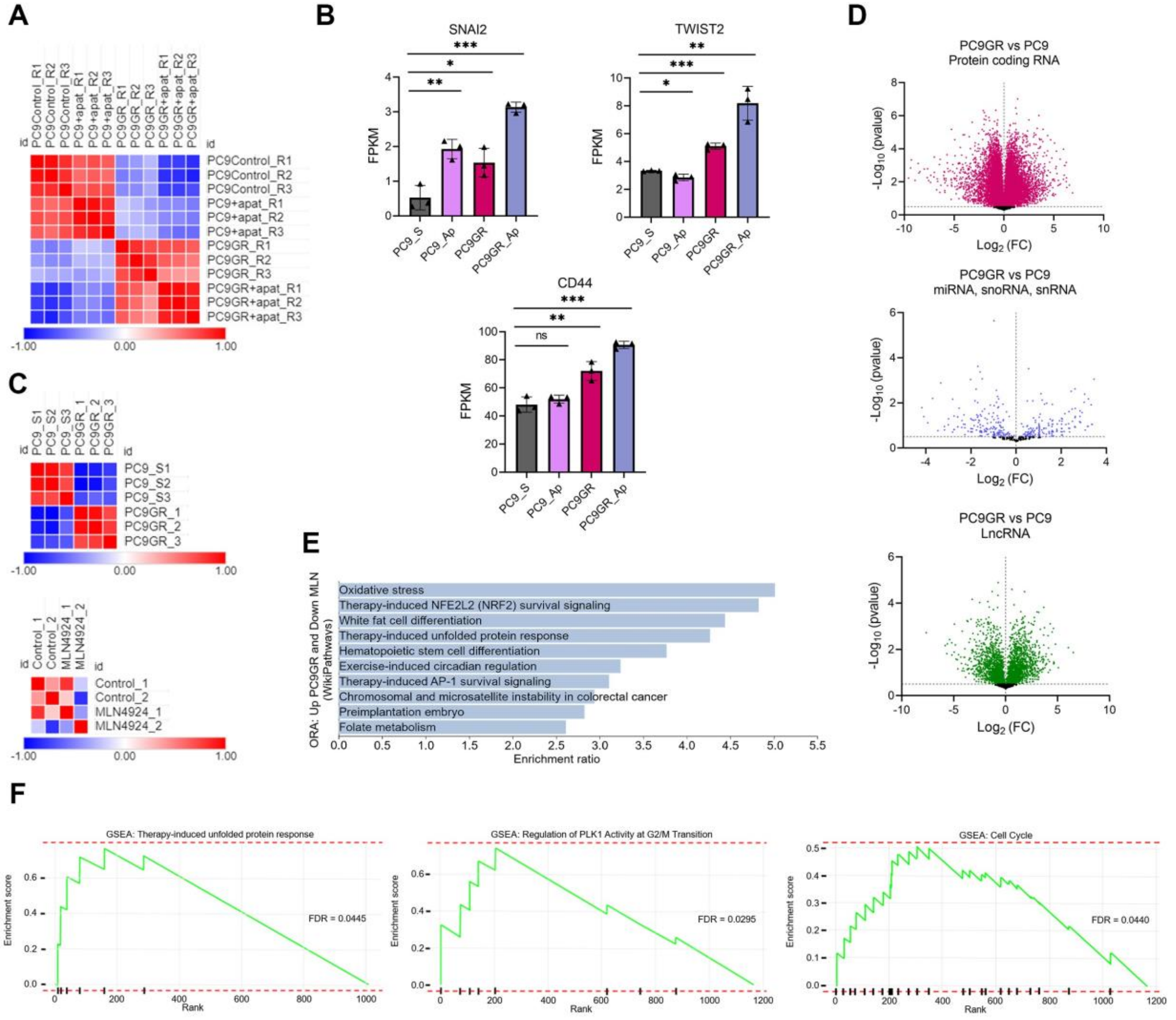

Supplementary Figure S3. The gene-expression profiles from tumorigenic chemosensitive and parental lung cancer cells. (A) Correlogram showing the correlation score matrix (FPKM values, Spearman/Kendall correlation coefficient) across RNA-seq data of PC9GR and their parental cells, both treated with apatinib. (B) Selected stemness-EMT target genes in PC9GR compared to the mean FPKM values in parental cells, and in both treated with Apatinib. Bar plots presenting data as means; error bars, s.e.m. (n = 3 biologically independent experiments); asterisks, P-values after One-way ANOVA, post hoc Dunnet: ***P ≤ 0.001; **P ≤ 0.01; *P ≤ 0.05. (C) Correlograms showing the correlation score matrix (FPKM values, Spearman/Kendall correlation coefficient) across RNA-seq data of PC9GR and parental cells (upper), and RNA-seq across RNA-seq data of lung A549 cancer cells treated with MLN4924 (down). (D) Volcano plot for differentially expressed mRNAs (pink), sncRNAs (blue), and lncRNAs (green) between PC9GR and parental cells. (E) Gene Set Enrichment Analysis (GSEA) of the upregulated transcripts in PC9GR, with complementary expression in upon MNL4924 treatmen. FDR, False discovery rate; FPKM, Fragments Per Kilobase per Million mapped fragments.
